# An Evaluation of the Intubrite Laryngoscope in Simulated In-Hospital and Out-of-Hospital Settings by Individuals with No Clinical Experience: A Randomized, Cross-Over, Manikin Study

**DOI:** 10.3390/diagnostics12071633

**Published:** 2022-07-05

**Authors:** Paweł Ratajczyk, Michał Fedorczak, Tomasz Gaszyński

**Affiliations:** Department of Anaesthesiology and Intensive, Therapy Medical University of Lodz, 90-419 Lodz, Poland; michal.fedorczak@umed.lodz.pl (M.F.); tomasz.gaszynski@umed.lodz.pl (T.G.)

**Keywords:** Macintosh laryngoscope, Intubrite laryngoscope, endotracheal intubation, simulation

## Abstract

Introduction: The aim of the study was to compare the Intubrite laryngoscope and the standard Macintosh blade laryngoscope (MCL) used by persons with no clinical experience in simulated hospital and non-hospital conditions on a manikin model. Materials and methods: The study involved 50 students of Medical Rescue. The hospital conditions (intubation height 110 cm—high position) and those occurring at the scene (intubation of a manikin located at floor level-low position) were simulated. The analysis included: duration of intubation, visibility of the laryngeal opening by the Cormack–Lehane scale, the bioelectrical activity of the intubating muscles, comfort and subjective assessment of physical effort by the Borg scale (Borg’s scale of subjective feeling of effort). The statistical analysis was performed with Microsoft Excel and T-student tests for pairs with unequal variables. The statistical importance was set at *p* < 0.05. Results: The use of an Intubrite laryngoscope significantly reduces the mean endotracheal intubation time compared to the Macintosh laryngoscope in a low position (17.34 s versus 19.04 s, *p* < 0.05). A higher rate of repeat intubations was observed in a low position for MCL (from 10% to 14%, *p* < 0.05). The reverse is true for Intubrite laryngoscope intubation (from 8% to 4%, *p* < 0.05 Please explain what is compared). The Intubrite laryngoscope improved visualization of glottis in the high and the low positions compared to the Macintosh laryngoscope (54% and 50% to 52% and 38%, respectively, *p* < 0.05). The risk of tooth damage was the same for the Intubrite and the MCL laryngoscopes in a high position (16% and 14%, respectively, *p* > 0.05), while in the simulated out-of-hospital setting, it was significantly higher for the MCL (22% versus 8%, *p* < 0.05). The subjective comfort of intubation in both simulated situations was similar according to the Borg scale (*p*-value, values). The use of the Intubrite laryngoscope was associated with less effort than the MCL in high versus low positions. For MCL, intubation in a high position was associated with lower mean muscle activity than in a low position (+48.24 µV versus +58.25 µV, *p*-value). For the Intubrite laryngoscope, these values were at similar levels (+52.03 µV and +52.65 µV, *p*-value). Conclusions: The use of the Intubrite laryngoscope by people with no clinical experience shortens the time of intubation and improves the laryngeal view compared to the standard Macintosh laryngoscope, but it requires similar muscle work in simulated conditions.

## 1. Introduction

Endotracheal intubation is the gold standard for airway management in patients with respiratory failure [1]. It enables the implementation of adequate ventilation, and it protects the lungs against aspiration [2]. The correct performance of endotracheal intubation requires not only theoretical knowledge but also considerable manual skills, which should be continuously improved [3]. Despite the above-mentioned requirements, there are patients with difficult airways, gastric content, or blood in the oral cavity or nasopharynx, whose intubation may be extremely difficult, resulting in tooth damage, soft tissue injury, CNS hypoxia, or cardiac arrest [2].

In the hospital setting, we have sets for difficult intubation that include various laryngoscopes, including video laryngoscopes, guidewires, supraglottic airway devices, and fiberscopes to reduce the risk of complications. Intubation is almost always performed on an operating table, hospital bed, or transport stretchers, the height of which can be adjusted according to our needs. In case of difficulty, we can ask someone to help us.

Contrary to hospital conditions in which a person intubates the patient from behind his/her head at a height adjusted to the needs of medical personnel, intubation of victims in the out-of-hospital setting is often carried out in conditions considered uncomfortable. This particularly concerns patients injured in road traffic accidents, work accidents, and situations related to mountain or cave rescue in which access to the patient in the standard position is difficult, impossible, or dangerous for the rescuer [4]. In the majority of cases, these are after trauma patients in whom the mobility of the cervical spine should be limited and who should be treated as full-stomach patients at increased risk of gastric content aspiration [2]. Such patients are intubated at the ground level, i.e., in a position extremely uncomfortable for the rescuer, often in unfavorable weather conditions. In the out-of-hospital setting, access to more advanced devices facilitating endotracheal intubation is also markedly limited.

All these factors influence the stress level of the person performing intubation, affecting the speed and the safety of the procedure and, consequently, the safety of the patient.

In most cases, classic Macintosh laryngoscopes are used to intubate patients.

**Figure 1:**
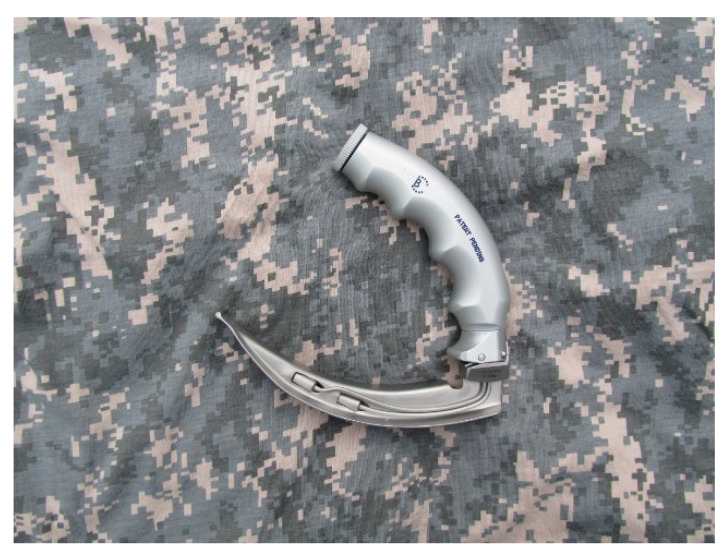
Intubrite laryngoscope (authors own material).

In this study, the feasibility of using an Intubrite laryngoscope (Intubrite^®^, LLC, Vista, CA, USA), which has a more ergonomic design than the classical laryngoscope (Figure 1), in simulated in-hospital and out-of-hospital settings by persons with little experience in endotracheal intubation was evaluated by comparing it to a Macintosh laryngoscope on a manikin model. The Intubrite laryngoscope is equipped with additional ultraviolet light, which might provide a better distinction between structures of entrance to the larynx and other tissues, especially in case of bleeding.

## 2. Material and Methods

The aim of the present study was to assess the feasibility of using the Intubrite laryngoscope in simulated hospital and non-hospital conditions by persons with no experience in endotracheal intubation and to compare it with the most commonly used Macintosh laryngoscope. The Intubrite laryngoscope (Intubrite^®^, LLC, Vista, CA, USA) has a more ergonomic shape due to a more contoured handle than a classic laryngoscope. This should allow for easier insertion of the laryngoscope into the patient’s mouth, requiring less force. The hospital conditions optimal for endotracheal intubation were simulated by placing the Laerdal Airway Management Trainer manikin of universal difficulty on the operating table at 110 cm; the non-hospital conditions were simulated by placing the manikin in a floor level-low position.

Fifty randomly selected students in the third year of full-time undergraduate medical education at the Medical University of Lodz were recruited for the study after obtaining the consent of the Bioethics Committee of the Medical University of Lodz. The students gave their informed consent to participate in this simulation study.

During the study, the following were assessed: the time required to perform successful endotracheal intubation with both laryngoscopes in high and low positions–primary outcome, the number of attempts, the subjective assessment of the degree of visualization of the laryngeal entry using the Cormack–Lehane scale (Cormack scale), the subjective assessment of striated muscle fatigue using the Borg scale (Borg’s scale of subjective feeling of effort), striated muscle activity using EMG, the number of early complications (tooth damage), the subjective assessment of comfort during endotracheal intubation, and the relation between subjective comfort and the number of early postintubation complications. The Borg scale with a range of 6–20 was replaced by a scale with a range of 0–10 where 0 meant complete lack of exertion and 10 meant extreme exertion [3]. The Cormack–Lehane scale is used to assess intubation difficulties when performing direct laryngoscopy. According to this scale, numerical grades indicate the following: 1-full view of glottis, 2-partial view of glottis, 3-only posterior extremity of glottis seen or only arytenoid cartilages, 4-only epiglottis seen and none of glottis seen, and 5-neither glottis nor epiglottis seen [3].

Each participant was familiarized with the simulation setting, the endotracheal intubation technique, the correct intubation position, and the construction and the functionality of the Macintosh laryngoscope and the Intubrite laryngoscope. After discussion of the study, participants were fitted with three disposable surface electrodes as required by the manufacturer of the EMG apparatus (ElectroMyoGraphy-EMG, MyoPlus2, Verity Medical, Tagoat, Ireland) to receive electrical action potentials of the left upper limb striated skeletal muscles. Electrode attachment sites were as follows:

1. Electrode I-the biceps brachii muscle (m. biceps brachii) [5].
2. Electrode II−1/3 proximal to the *brachioradialis* muscle (*m. brachioradialis)* [3].
3. Electrode III-grounding electrode-ulnar process surface *(olecranon)* [3].

After proper placement of the electrodes, the participant was asked to rest for 2 min during which deliberate tensing of the upper limb muscles was avoided.

The participant’s task was to correctly perform four endotracheal intubations of two series performed on a Laerdal manikin (Laerdal Airway Management Trainer) of universal difficulty for endotracheal intubation.

Each series consisted of performing two correct endotracheal intubations. The first endotracheal intubation was performed using the Macintosh laryngoscope and the subsequent used the Intubrite laryngoscope. Between both intubations, the participant was obliged to take a 2-min break to recover muscle strength and tension. The first series was performed in conditions imitating hospital conditions, i.e., the manikin was placed at the height of 110 cm. The second series was performed under conditions simulating those in non-hospital conditions, i.e., the manikin was placed at the floor level. Between sets, a mandatory 2-min rest was taken to regenerate strength and muscle tone.

During intubation, the timing of the intubation was measured using a sports stopwatch. Time measurement began with the “start” command after confirmation of the participant’s preparation for intubation, and time was measured until the intubation tube was correctly confirmed. If intubation was not successful, the participant immediately proceeded to the next attempt.

After all endotracheal intubations were performed, the participant was removed from the EMG machine electrodes and asked to complete an anonymous questionnaire about the study. The questionnaire consisted of questions regarding the degree of visualization of the laryngeal entry, comfort, and subjective assessment of the difficulty of intubation. The study also evaluated the possibility of tooth damage during intubation. This risk is directly related to many factors, i.e., inadequate visualization of the laryngeal opening, the participant’s poor grip on the laryngoscope, or a lack of muscle strength [5]. 

The statistical analysis was performed with Microsoft Excel and T-student tests were used for pairs with unequal variables. The statistical importance was set at *p* < 0.05. The sample size was calculated using https://clincalc.com/stats/samplesize.aspx (accessed on 25 January 2016). We estimated the reduction in time necessary to intubate using Intubrite in a low position by 20% compared to a standard Macintosh blade laryngoscope as the primary outcome measurement. Based on a power of 80% and on setting the importance of the *p* value on a level of 0.05, the necessary group size was calculated on 38. The size of the group, 50, was sufficient for detecting a decrease in time to intubation.

## 3. Results

The analysis of the results obtained in the study began with the appropriate grouping of data obtained from direct measurements and observations as well as data obtained from the questionnaire. Then, the information obtained was processed for the work in Microsoft Excel 2007.

**Figure 2:**
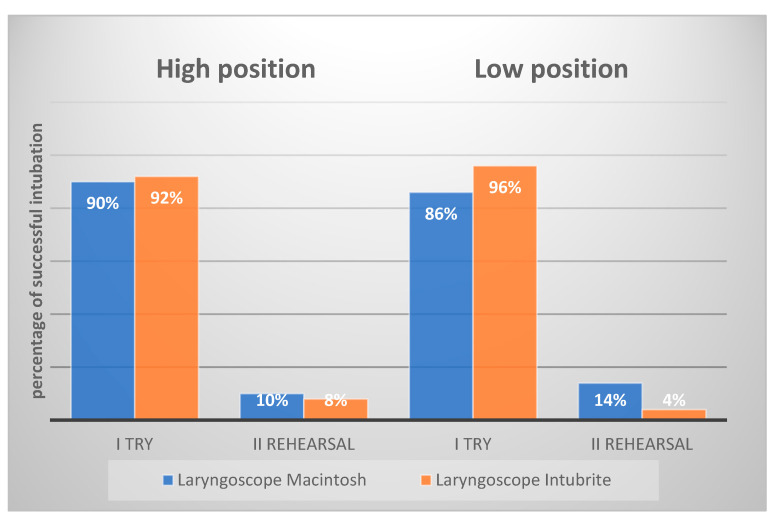
Graphical chart of the success rate of intubation.

The first parameter analyzed was the time needed for proper endotracheal intubation. The use of Intubrite laryngoscopes in out-of-hospital conditions shortened the meantime to successful tracheal intubation. The average intubation time was significantly lower for Intubrite vs. MCL in a low position (16.86 vs. 19.04, *p* < 0.05) but not in a standard position (Table 1, Figure 2).

Using the Macintosh laryngoscope, intubation at floor level, compared to intubation in the high position, was associated with an increased risk of intubation failure from 10% to 14% (*p*-value). The opposite was observed with the Intubrite laryngoscope, where intubation in the low position reduced the risk of repeated intubation to 4% from the described 8% in the high position (*p* < 0.05).

Based on the data, the use of the Macintosh laryngoscope in the simulated out-of-hospital setting is associated with more than three times the risk of intubation failure compared with the Intubrite laryngoscope (from 14% to 4%). Thus, in simulated conditions, the use of MCL was associated with more than three times the number of attempted failures (14% compared to 4%, *p*-value).

**Figure 3:**
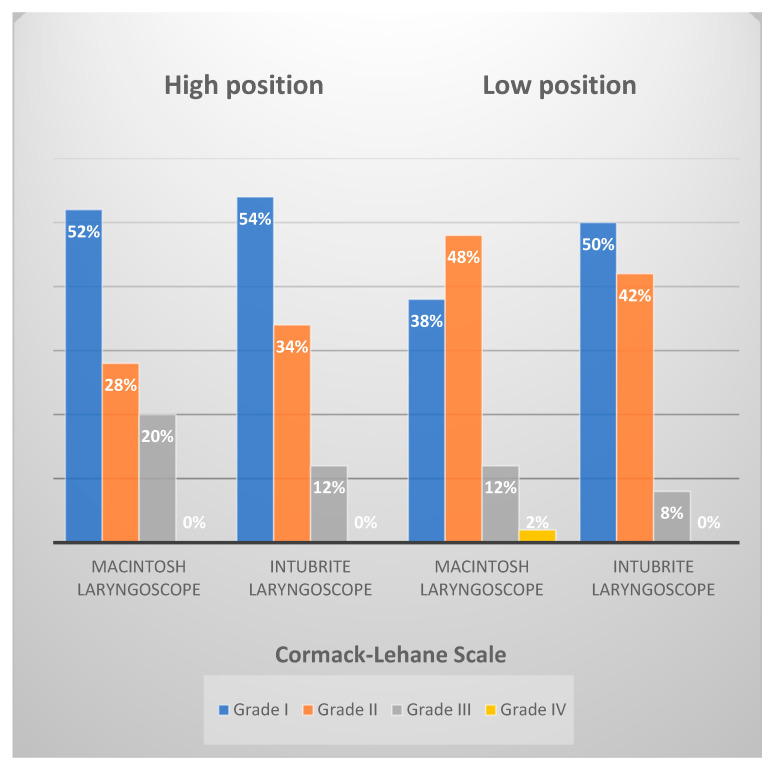
Percentage distribution of laryngeal entry visualization according to the Cormack–Lehane scale.

Based on the data obtained, a high degree of visualization of the laryngeal entry during endotracheal intubation was observed with both studied laryngoscopes (Figure 3). The Intubrite laryngoscope provided relatively better visualization of the vocal cords during intubation compared with the Macintosh laryngoscope in in-hospital and out-of-hospital settings (*p* < 0.05) (t-student test, comparison of Cormack–Lehane scale obtained by Intubrite or MCL). In more than 50% of three simulated situations (high position of the Macintosh laryngoscope and Intubrite and low position of the laryngoscope Intubrite), full visibility of the glottis was achieved In 38% of cases, the best visibility of the airway was obtained when using the Macintosh laryngoscope in the low position.

It is worth noting that one student indicated that intubation was performed without seeing the vocal cords.

**Figure 4:**
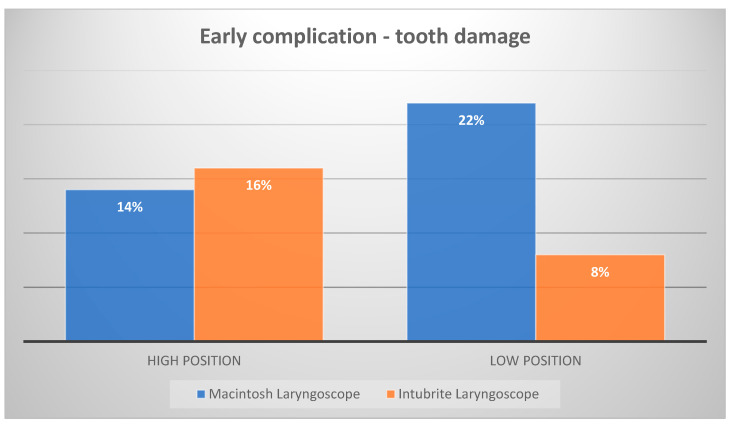
Presence of tooth damage during intubation.

The data showed that intubation in the simulated out-of-hospital setting performed at floor level compared to the high position increased the risk of tooth damage when using the classic laryngoscope from 14% to 22% (*p* < 0.05, t-student test for pairs with unequal variations) (Figure 4). The use of an Intubrite laryngoscope with a specially shaped handle for intubation under the same conditions resulted in a 50% reduction in the risk of tooth damage compared to the situation simulating hospital conditions from 16% to 48%. When comparing the two laryngoscopes, there appeared to be little difference in the assessment of possible injuries under simulated hospital conditions. For intubation in the simulated out-of-hospital setting, the use of the Intubrite laryngoscope results in a reduction in possible tooth injury to 8% compared with 22% of episodes associated with the use of the Macintosh laryngoscope (*p* < 0.05, t-student test for pairs with unequal variations).

**Figure 5:**
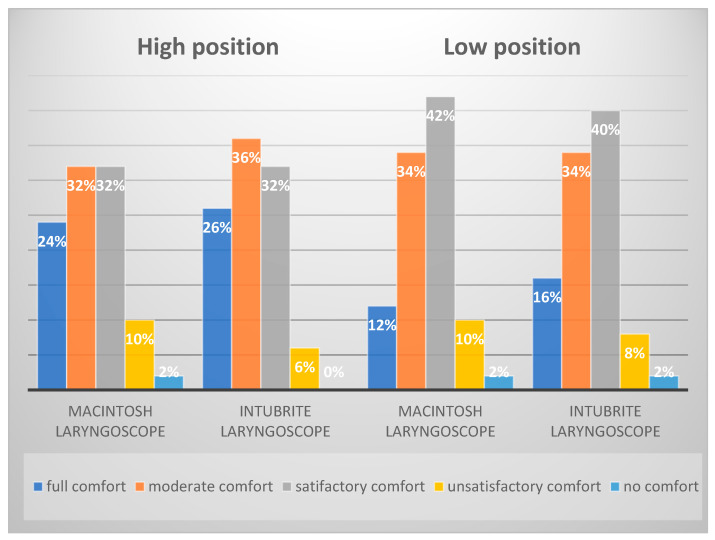
Assessment of the comfort of intubation with the Macintosh and the Intubrite laryngoscopes depending on the simulation conditions.

No substantial differences in comfort were observed for both the positions and the types of laryngoscopes used (Figure 5). The prevailing opinion of respondents was satisfactory: 32% of opinions during intubation in conditions simulating hospital conditions using both laryngoscopes and 40% of opinions during intubation in conditions similar to those in out-of-hospital settings with the Macintosh laryngoscope.

In cases of simulated in-hospital conditions, 80% of respondents (40 opinions) believed that endotracheal intubation with the Intubrite laryngoscope was associated with moderate or less exertion. Moreover, the laryngoscope with a profiled handle received more opinions confirming the lack of subjective feeling of effort-5 opinions (Macintosh laryngoscope-2 opinions). For the Macintosh laryngoscope, 70% of respondents (35 opinions) stated that endotracheal intubation required moderate or less effort. In the case of both laryngoscopes, none of the subjects during endotracheal intubation indicated that the activity required effort described as very heavy or extremely heavy.

The simulated hospital conditions were similar during intubation with an Intubrite laryngoscope in simulated non-hospital settings. Here again, 80% of respondents (40 persons) thought that intubation should be performed with moderate or less effort. Use of the Macintosh laryngoscope in these conditions is associated with effort described as moderate or less in 64% of subjects (32 opinions). In the case of both laryngoscopes, none of the subjects, during endotracheal intubation in these conditions, indicated that this activity required effort defined as very heavy or extremely heavy.

Objective elements assessing the effort during intubation include muscle tension involved in this action. The study involved the two most important muscles responsible for the movement at the elbow and the radiocarpal joints, i.e., biceps brachii and radialis brachii.

When using a Macintosh laryngoscope, an increase in muscle activity was observed from 48.24 µV to 58.25 µV when changing the intubation position: from high to low (*p* < 0.05). When intubating with the Intubrite laryngoscope, the difference in muscle tension was minimal (*p* > 0.05). Comparing both laryngoscopes, the use of a Macintosh laryngoscope was associated with lower muscle activity, whereas the opposite is true in non-hospital settings. 

## 4. Discussion

Securing an unobstructed airway and proper ventilation of the patient is the basic determinant of survival of patients with respiratory failure in emergency medicine or intensive therapy [3]. This problem has become very important in the era of the SARS COVID-19 virus pandemic, during which lower respiratory tract symptoms were one of the first signs of general deterioration [5]. A marked increase in the number of patients with the occurrence or severity of respiratory insufficiency forced increased concern about the ventilatory safety of patients, also among medical personnel, who had not dealt with such patients in their everyday work [6].

The best way to secure an airway is endotracheal intubation [1,7]. However, this action is not simple and requires constant training as over the years it can be forgotten, particularly by persons who do not perform it every day [8]. The degree of endotracheal intubation failure is particularly high in novices using classical laryngoscopes with Macintosh handles and much lower in those using video laryngoscopes [9]. Video laryngoscopes increase the effectiveness of intubation, particularly in patients with difficult airways, improve the visibility of the glottis, and it may reduce laryngeal and airway trauma [10,11]. However, there is currently no evidence that they reduce the number of intubation attempts or the incidence of hypoxia [2]. Moreover, there is no evidence that the use of video laryngoscopes affects the required time of intubation [1]. Hodd and Reutzler found that there was no advantage of video laryngoscopes during intubation of patients with normal airways; only in patients with difficult airways did they affect the speed of intubation [8,12].

The problem of maintaining effective ventilation is even more difficult when access to the victim’s head is limited, the victim is lying on the floor, or the alignment of the oral cavity, pharynx, and trachea, preferably enabling visualization of vocal cords, is difficult [13]. In such situations, the victim’s body is often in a non-physiological position in which various muscle parts are tensed, causing considerable discomfort [3]. Additionally, the stress related to the patient’s condition, time pressure, and responsibility for the patient’s life and health increase the sense of discomfort [6].

In a situation where the patient has been infected with the COVID-19 virus, the sense of discomfort in the rescuer, wearing personal protective equipment with an FFP-3 mask and a face shield, is further increased [6]. Under such conditions, the possibility of ineffective or prolonged intubation or causing intubation complications in the patient increases markedly. The use of video laryngoscopes by paramedics wearing protective clothes improves the speed of intubation compared to the classical Macintosh laryngoscope [6]. Other conclusions were reached by Yousif who found that the use of video laryngoscopes by paramedics dressed in protective equipment and having little experience in intubation did not affect the speed of intubation compared to the Macintosh laryngoscope [5].

The use of classical Macintosh laryngoscopes by non-experienced rescuers may result in prolonged intubation, intubation complications, or even death of the patient [9]. The use of video laryngoscopes, which greatly facilitates endotracheal intubation in this group of medical personnel, is sometimes excluded due to the limited availability of such devices, including financial reasons [14]. Therefore, one should look for a laryngoscope that would be more friendly to inexperienced intubators and cause less complications than the classical Macintosh laryngoscope.

In the present study, the feasibility of using the Intubrite laryngoscope simulated in-hospital (manikin height 110 cm) and out-of-hospital conditions (manikin lying on the floor) by third-year students of emergency medicine, i.e., those with no clinical intubation experience, was assessed and compared to the classical Macintosh laryngoscope [15].

When the Intubrite laryngoscope was used, simulated in-hospital (manikin lying at 110 cm) and out-of-hospital (manikin lying on the floor) intubation was found to require, on average, the same amount of time. Comparing the two laryngoscopes, it can be said that the use of the Intubrite laryngoscope in simulated out-of-hospital settings reduces intubation time by an average of 2.54 s. compared to the Macintosh laryngoscope. Moreover, in cases when the Intubrite laryngoscope is used in simulated out-of-hospital conditions, the percentage of repeated intubation attempts and tooth damage is lower compared to the Macintosh laryngoscope; the results are similar to those of J. Tesler and J. Rucker [4]. Similar results were obtained by Tomasz M. Gaszyński, who stated that the Intubrite laryngoscope is less traumatic to patients than the Macintosh laryngoscope [3]. On the other hand, Szarpak and co-workers found that in difficult airways (tongue edema), the time needed for intubation by novice physicians in manikin tests was similar for both laryngoscopes, although the effectiveness of the first intubation was higher for Macintosh laryngoscopes [16].

One of the basic conditions for successful tracheal intubation is a visual observation of the passage of the endotracheal tube through the vocal cords. Our study demonstrated a high degree of laryngeal entry visibility with both laryngoscopes tested in both in-hospital and out-of-hospital conditions. Under non-hospital conditions, the Intubrite laryngoscope was better as it enabled half of the study participants to visualize the laryngeal opening fully (grade I according to the Cormack—Lehane scale) (the Macintosh laryngoscope enabled this in 38% of study participants). Similar results concerning the Macintosh laryngoscope were obtained by Wolfgang A. Wetsch, who compared the usefulness of Macintosh laryngoscopes and videoscopes during endotracheal intubation in uncomfortable conditions with markedly limited access to the victim’s head [17].

In our study, comfort during intubation in a given position and with a given laryngoscope was considered a reliable source of information about the degree of difficulty and usefulness of the equipment used [3]. The data analysis demonstrated that intubation with a Macintosh laryngoscope and a newer generation Intubrite laryngoscope was equally comfortable both in simulated hospital and out-of-hospital conditions. The study did not demonstrate a clear benefit of using a given laryngoscope in terms of comfort.

Muscle fatigue during tracheal intubation can be a significant problem, especially during direct laryngoscopies associated with difficult airways. The introduction of more ergonomic devices would reduce the burden on the rescuer, which is an important factor for patient safety [3]. This is particularly true for persons with little experience in intubation, in whom potential intubation difficulties are more common, especially in obese patients; due to their body build and airway anatomy, such patients may require high forces to clear the airway [3]. Therefore, the person should use a device that does not require much effort during intubation. Using EMG, it was found that the use of a Macintosh laryngoscope in the low position required more skeletal muscle work compared to intubation in the hospital setting (58.25 µV to 48.24 µV, *p*-value). When the Intubrite laryngoscope was used, the average muscle activity was at the same level in the in-hospital and out-of-hospital settings. The use of the Macintosh laryngoscope in hospital conditions (high position) is associated with lower skeletal muscle activity than the use of the Intubrite laryngoscope (48.24 µV to 52.65 µV, *p*-value). Similar conclusions were reached by Tomasz Gaszyński and Jakub Jakubiak, who compared muscle activity during intubation of patients with Macintosh and Intubrite laryngoscopes and video laryngoscopes in operating room conditions, i.e., in the high position. They did not study one use of laryngoscopes in the out-of-hospital setting (low position). In our study, when it is necessary to intubate in the out-of-hospital setting the use of an Intubrite laryngoscope is associated with less muscle activity than the use of a Macintosh laryngoscope (52.65 µV to 58.25 µV, *p*-value). This is probably related to the more ergonomic shape of the Intubrite laryngoscope. If intubation had to be repeated, increased muscle activity was found only when intubating with the Macintosh laryngoscope in the low position. When the relationship between the incidence of early intubation complications and increased muscle activity was assessed, such a correlation was observed when the Intubrite laryngoscope was used in hospitals and the Macintosh laryngoscope in out-of-hospital settings. Gaszyński and Jakubowski did not demonstrate any relation between increased muscle tone and the incidence of trauma in the intubated patient. This might have been related to the relatively small study group of 13 young anesthesiology residents [3].

Assessing the degree of effort involved in airway management using the Borg subjective effort scale, it was found that the use of an Intubrite laryngoscope was associated with a lower subjective perception of the physical effort involved in endotracheal intubation. This applies both to intubations in conditions simulating hospital conditions and those referring to conditions occurring at the scene of an accident.

The study also demonstrated the necessity of continuous training in airway management, including endotracheal intubation [18]. Learning how to deal with unconventional situations requiring modifications in technique, equipment, or position is particularly valuable [7,19]. Every exercise in this field reduces the risk of errors, decreases the stress of personnel performing a given procedure and, more importantly, increases the chances of survival of victims and their return to the pre-incident state [2].

The study limitation may be the simulation model; however, it may also be considered as a study strength: since the evaluation is performed in a simulation laboratory, there are no risks for real patients. Especially clinicians with no clinical experience were involved. Novices indeed are to be studied, as complications are associated with a lack of experience and proficiency is associated with higher success rates. From an ethical point of view, this kind of study is the optimal way to first identify the benefits of one technique over another. We feel that there is importance in evaluating new devices or techniques in a simulation setting. It is not ethical to investigate a new device used by novices in real-life. This is why simulation studies need to be conducted first. This is a positive justification for the study. However, it must be underlined that simulation is not equal to clinical practice and that further research studies involving these devices need to be conducted.

Based on the results obtained, the use of Intubrite laryngoscopes for intubation of patients in simulated in-hospital and out-of-hospital settings seems to be more beneficial than Macintosh laryngoscopes, particularly in the out-of-hospital setting (low position). The above statement needs to be confirmed in studies with a larger study group, especially in humans. Transferring the results of manikin studies into real-life should be done carefully, but the manikin studies can be an introduction to research on new devices, and based on the results of manikin studies the human studies can be better designed and planned.

## 5. Conclusions

The use of the Intubrite laryngoscope for intubating patients in simulated inpatient and out-of-hospital settings by people with no clinical experience shortens the time of intubation and improves the laryngeal view, when compared to a standard Macintosh laryngoscope, but it requires similar muscle work.

## Figures and Tables

**Table 1:** Time required to intubate manikin in the high and the low positions with Macintosh and Intubrite laryngoscopes.

Obtained Time [s]	High Position		Low Position	
	Macintosh Laryngoscope	Intubrite Laryngoscope	Macintosh Laryngoscope	Intubrite Laryngoscope
Average	17.34	16.9	19.04	16.86
Median	15	15	18	15
Modal	15	8	17	8
Minimum	7	7	7	8
Maximum	39	45	47	38
Variance samples	74.54	62.93	71.83	60.86
Deviation standard	8.72	8.01	8.47	7.80
Skewness	0.26	1.11	0.24	1.13
*p* value	>0.05		<0.05	

## Data Availability

Data Available by authors on request.
